# Association between obesity and medical expenditures among Japanese adults treated for diabetes: A secondary analysis

**DOI:** 10.1371/journal.pone.0349416

**Published:** 2026-05-19

**Authors:** Yuki Yonekura, Kozo Tanno, Aya Higashiyama, Nagako Okuda, Tomomi Nagahata, Akira Okayama

**Affiliations:** 1 Department of Nursing Informatics, Graduate School of Nursing Science, St Luke’s International University, Chuo-ku, Tokyo, Japan; 2 Department of Hygiene and Preventive Medicine, Iwate Medical University, Yahaba-cho, Iwate, Japan; 3 Department of Hygiene, Wakayama Medical University, Wakayama, Wakayama, Japan; 4 Division of Applied Life Sciences, Graduate School of Life and Environmental Sciences, Kyoto Prefectural University, Kyoto, Kyoto, Japan; 5 Research Institute of Strategy for Prevention, Chuo-ku, Tokyo, Japan; Aomori University, JAPAN

## Abstract

In Japan, where obesity prevalence is lower than in Western countries, few studies have examined the association between obesity and medical expenditures among patients with diabetes, distinguishing between overweight and obesity by sex. This study examined the association between obesity and medical expenditures among patients with diabetes in Japan. Data on medical expenditures and the Specific Health Checkups obtained from 12 municipal and six union insurers for fiscal years 2008 and 2009 were analyzed. Gamma regression and quantile regression were used to compare annual total, outpatient, and inpatient medical expenditures in fiscal year 2009 among three groups: normal/underweight (body mass index [BMI] < 25 kg/m^2^), overweight (BMI: 25–30 kg/m^2^), and obesity (BMI ≥ 30 kg/m^2^). The analyses were performed separately for men and women, adjusting for age, hypertension, hyper-low-density lipoprotein cholesterolemia, glycemic control, and smoking. Logistic regression was performed, adjusting for the same variables to assess the occurrence of annual total medical expenditures of ¥1 million (approximately US$10,600 in 2009) or more. Annual total medical expenditures were significantly higher in the obesity group than in the normal/underweight group among both men and women (exp(β) = 1.117 [95% confidence interval (CI): 1.023–1.221] among men and exp(β) = 1.157 [1.036–1.295] among women). Both men and women in the overweight and obesity groups had significantly higher outpatient expenditures than those in the normal/underweight group. The overweight and obesity groups had significantly higher medical expenditures than the normal/underweight group in most quantiles among men. The likelihood of annual total medical expenditures exceeding ¥1 million was higher among women with obesity than among those with normal/underweight (odds ratio = 1.546 [95% CI: 1.007–2.375], *p* = 0.047). These findings suggest that medical expenditures among patients with diabetes who have overweight or obesity are higher than those of normal/underweight patients.

## Introduction

In Japan, 18.1% of men and 9.1% of women aged ≥ 20 years have a hemoglobin A1c (HbA1c) level of ≥ 6.5% or are receiving treatment for diabetes [1]. Moreover, the number of “persons strongly suspected to have diabetes” is estimated to be approximately 14 million [1]. Medical expenditures related to diabetes in Japan totaled JPY (¥) 1,199.4 billion (approximately US$10.8 billion in 2021), accounting for 3.7% of the total medical expenditures in 2021 [2]. Diabetes is also a risk factor for diseases that account for a large proportion of medical expenditures, such as cardiovascular diseases, which cost ¥6,111.6 billion (approximately US$55.2 billion in 2021) annually and account for 18.9% of the total medical expenditures, and kidney diseases (including glomerular diseases, tubulointerstitial kidney diseases, and renal failure), which cost ¥1,632.9 billion (approximately US$14.7 billion in 2021) annually and account for 5.0% of the total medical expenditures.

Obesity is strongly associated with diabetes, and an increased body mass index (BMI) is correlated with a higher frequency of diabetes [3,4]. Among patients with diabetes, individuals with obesity exhibit an elevated rate of poorly controlled diabetes and an increased frequency of complications [5]. Moreover, the presence of obesity-related diabetes complications is associated with increased rates of hypertension-related complications and increased medical expenditures. Specifically, poorly controlled diabetes mellitus often leads to chronic complications, such as chronic kidney disease, resulting in expensive medical care. Notably, medical expenditures among patients with diabetes and obesity are higher than those among patients without obesity [6–9].

The association between diabetes control rates, medical expenditures, and obesity among patients receiving diabetes treatment in the general Japanese population has rarely been reported. This is because, in Japan, there are fewer people classified as having obesity (BMI ≥ 30 kg/m^2^) compared to those in Western countries. In 2022, the estimated proportion of individuals with a BMI of ≥ 30 kg/m^2^ in Japan was 7.5% for men and 3.5% for women aged 18 years and older. In comparison, the figures are 31.0% for men and 36.5% for women in the Americas, and 22.3% for men and 22.7% for women in Europe [10]. A previous report on medical expenditures among patients with diabetes indicated average medical expenditures of approximately ¥270,000 (approximately US$2,450 in 2018) per year, with increased medical expenditures being associated with higher BMI categories [7]. Although this previous study provided a detailed examination of medical expenditures by outpatient, inpatient, dispensing, and medication types, it had limitations including a single-center design, lack of classification for individuals with a BMI of ≥ 30 kg/m^2^, and lack of multivariable adjustment.

Therefore, the present study aimed to determine the cost of obesity by analyzing the characteristics of individuals undergoing diabetes treatment according to BMI categories, including examination results and medical expenditures.

## Materials and methods

### Study design and population

This study is a secondary analysis of existing data. Data from a previous study, “Study on the Influence of Specific Health Checkups and Specific Health Guidance by Medical Insurers on Medical Expenses (MHLW H20-Policy, General-014),” were analyzed.

The primary data collection was conducted from 2009 to 2010. As data on medical expenditures and the Specific Health Checkups were accumulated for business purposes and owing to the considerable difficulty in obtaining retrospective consent from individually insured persons, consent was not obtained from the insured persons [11]. Consequently, anonymized data were collected. The data collection procedures complied with the “Ethical Guidelines for Epidemiological Research” in Japan at that time, and permission was obtained from the ethics committee of the Japan Anti-Tuberculosis Association, the institution to which the principal investigator of the research group belonged. After obtaining approval from the institutional ethics committee of St. Luke’s International University (Approval number: 23-A094), approval was sought from the secretariat of the research group to use the data, and permission was granted on December 21, 2023. The authors did not have access to information that could identify individual participants during or after data collection.

Participants who met the following conditions were included in the analysis: (1) responded “taking medication for diabetes (including oral medications and insulin)” in the questionnaire of the Specific Health Checkups; (2) had measurements of BMI, fasting blood glucose, or HbA1c during the Specific Health Checkups; (3) had medical expenditures in fiscal year (FY) 2008 of less than ¥1 million (approximately US$10,600 in 2009); (4) were not hospitalized in FY2008; (5) were aged between 40 and 74 years in FY2008; and (6) had valid data for use in the analysis, including age, sex, treatment status (hypertension, diabetes, and hyper-low-density lipoprotein (LDL) cholesterolemia), smoking status, systolic blood pressure, diastolic blood pressure, LDL cholesterol, and measures for evaluating glycemic control (HbA1c or fasting blood glucose).

### Measures

#### Medical expenditures.

Medical expenditures were based on monthly medical expenditures data (receipt data) among participants from FY2007 to FY2009, including outpatient (sum of expenditures of treatment, pharmacy and lab tests), inpatient, and total of these medical expenditures, with 1 point equaling ¥10. The points were multiplied by 10, converted to monetary values, and converted to units of ¥1,000 (approximately US$10.6 in 2009) for the analysis.

High medical expenditures were defined as an annual total of 100,000 points (¥1 million: approximately US$10,600 in 2009) or more. This is because it is a convenient value corresponding to the top 5% of the subjects analyzed.

#### Other characteristics.

Data from the Specific Health Checkups in FY2008 were used. Participants were classified into the following groups based on the definition of the World Health Organization [12]: normal/underweight (BMI < 25 kg/m^2^), overweight (25 ≤ BMI < 30 kg/m^2^), or obesity (BMI ≥ 30 kg/m^2^). Glycemic control was considered poor if the fasting blood glucose level was ≥ 140 mg/dL or the HbA1c level was ≥ 7.0%. Hypertension was classified as present if the systolic blood pressure was ≥ 140 mmHg, the diastolic blood pressure was ≥ 90 mmHg, or if the patient was taking antihypertensive medication. Patients with LDL cholesterol levels ≥ 120 mg/dL or those taking cholesterol-lowering medications were considered to have hyper-LDL cholesterolemia based on the Japan Atherosclerosis Society Guidelines for Prevention of Atherosclerotic Cardiovascular Diseases 2022 [13].

### Statistical analysis

Participant characteristics were compared and tested by BMI categories. The Kruskal–Wallis test was employed for continuous variables, and the chi-squared test was utilized for categorical variables.

Because the distribution of medical expenditures is highly skewed and heteroscedastic, the association between medical expenditures and obesity among patients with diabetes was analyzed using gamma regression with a log-link function [14]. The outcome variables were annual total medical expenditures, annual outpatient expenditures, and annual inpatient expenditures in FY2009; the explanatory variable was BMI category; and the control variables included age, current smoking, glycemic control, hypertension, hyper-LDL cholesterolemia, and the mean annual medical expenditures for FY2007 and FY2008. First, a model adjusted for age alone was estimated (Model 1). Subsequently, a multivariable-adjusted model was developed, accounting for all the control variables above (Model 2). Given the large proportion of zero inpatient expenditures (approximately 90%), a zero-inflated gamma model was used to account for excess zeros (no hospitalization) and to model non-zero inpatient expenditures.

Quantile regression [15] was performed on annual total medical expenditures at the 10th, 25th, 50th, 75th, and 90th percentiles to assess the association between medical expenditures and BMI categories. BMI category was the main explanatory variable, and covariates included age, current smoking, glycemic control, hypertension, hyper-LDL cholesterolemia, mean medical expenditures in FY2007 and FY2008, and change in medical expenditures from FY2007 to FY2008. Excess medical expenditures for the overweight and obesity groups, compared with the normal/underweight group, were estimated based on these models. Quantile regression was selected because the association between medical expenditures and BMI varied across different quantiles of medical expenditures [8]. This approach enables estimation of associations at different points of the outcome distribution and is robust to outliers [15].

The association between high medical expenditures (≥ ¥1 million) and BMI categories among patients with diabetes was analyzed using logistic regression. BMI category was the main explanatory variable, and covariates included age, current smoking, glycemic control, hypertension, hyper-LDL cholesterolemia, and mean medical expenditures in FY2007 and FY2008.

Although alcohol intake, exercise, and diet are known risk factors, they were not included in the main analysis because they were not mandatory during the Specific Health Checkups and were missing for approximately 30% of the participants. For sensitivity analysis, drinking and exercise were included as control variables.

Furthermore, we used Oster’s method [16] to assess whether the estimated coefficients for the association between medical expenditures and obesity levels were robust to bias from unobserved confounders. A δ value greater than 1 indicates robustness to the effects of unobserved variables. Following Oster’s recommendation, we set R_max_ as 1.3 times the R^2^ value from a model including all explanatory variables used and calculated δ accordingly.

All analyses were performed separately for men and women, and the significance level was set at 5%. The analysis was performed using R ver. 4.4.0 (R Foundation, Vienna, Austria).

## Results

### Characteristics of the participants

The characteristics of the participants according to BMI category are presented in Table 1. Among the 15,833 men included in this study, 8,808 (55.6%), 5,574 (35.2%), and 1,451 (9.2%) were included in the normal/underweight, overweight, and obesity groups, respectively. Among 4,760 women, 2,638 (55.4%), 1,492 (31.3%), and 630 (13.2%) were categorized into the normal/underweight, overweight, and obesity groups. Compared to those with lower BMI, both men and women with higher BMI were younger, more likely to be affected by hypertension and hyper-LDL cholesterolemia, and significantly more likely to have poor glycemic control with fasting blood glucose levels of 140 mg/dL or higher or an HbA1c level of 7.0% or higher. The mean annual medical expenditures in FY2007 and FY2008 were also significantly higher in the group with higher BMI than in that with lower BMI, and the same trend was observed for the annual total medical expenditures and annual outpatient expenditures including medications in FY2009.

**Table 1:** Participant characteristics by BMI categories.

	Normal/underweight	Overweight	Obesity	p-value
Male				
Number of participants	8,808	5,574	1,451	
Age, years, median (Q1, Q3)	59 (54, 63)	58 (52, 62)	53 (47, 59)	<0.001^a^
Current smoker, n (%)	4,218 (48)	2,353 (42)	612 (42)	<0.001^b^
Poor glycemic control^c^, n (%)	4,711 (53)	3,071 (55)	837 (58)	0.006^b^
Hypertension^d^, n (%)	4,227 (48)	3,578 (64)	1,105 (76)	<0.001^b^
Hyper-LDL cholesterolemia^e^, n (%)	5,079 (58)	3,854 (69)	1,088 (75)	<0.001^b^
Mean annual medical expenditures FY2007–FY2008 (¥1,000), Median (Q1, Q3)	218 (134, 338)	239 (157, 355)	265 (174, 387)	<0.001^a^
Change in annual medical expenditures from FY2007 to FY2008 (¥1,000), Median (Q1, Q3)	13 (−33, 75)	12 (−39, 77)	19 (−33, 87)	0.21^a^
Total medical expenditures in FY2009 (¥1,000), Median (Q1, Q3)	228 (130, 371)	254 (156, 401)	287 (181, 441)	<0.001^a^
10th percentile	36	61	81	
25th percentile	130	156	181	
50th percentile (median)	228	254	287	
75th percentile	371	401	441	
90th percentile	603	658	666	
Annual outpatient expenditures in FY2009 (¥1,000), Median (Q1, Q3)	218 (127, 340)	246 (152, 367)	277 (176, 409)	<0.001^a^
Hospitalization in FY2009, n (%)	891 (10)	600 (11)	162 (11)	0.30^b^
Annual inpatient expenditures in FY2009 (¥1,000), Median (Q1, Q3)^f^	428 (168, 1,031)	422 (171, 1,064)	373 (178, 896)	0.72^a^
Total medical expenditures ≥ ¥1 million in FY2009, n (%)	375 (4.3)	270 (4.8)	71 (4.9)	0.20^b^
Female				
Number of participants	2,638	1,492	630	
Age, years, median (Q1, Q3)	60 (55, 65)	59 (54, 65)	56 (50, 62)	<0.001^a^
Current smoker, n (%)	314 (12)	189 (13)	98 (16)	0.046^b^
Poor glycemic control^c^, n (%)	1,151 (44)	751 (50)	334 (53)	<0.001^b^
Hypertension^d^, n (%)	1,240 (47)	986 (66)	485 (77)	<0.001^b^
Hyper-LDL cholesterolemia^e^, n (%)	1,958 (74)	1,203 (81)	527 (84)	<0.001^b^
Mean annual medical expenditures FY2007–FY2008 (¥1,000), Median (Q1, Q3)	230 (139, 347)	265 (175, 388)	281 (181, 414)	<0.001^a^
Change in annual medical expenditures from FY2007 to FY2008 (¥1,000), Median (Q1, Q3)	7 (−39, 64)	13 (−39, 77)	15 (−42, 92)	0.12^a^
Total medical expenditures in FY2009 (¥1,000), Median (Q1, Q3)	235 (134, 385)	278 (171, 426)	290 (188, 475)	<0.001^a^
10th percentile	30	78	99	
25th percentile	134	171	188	
50th percentile (median)	235	278	290	
75th percentile	385	426	475	
90th percentile	599	665	709	
Annual outpatient expenditures in FY2009 (¥1,000), Median (Q1, Q3)	229 (131, 362)	272 (169, 400)	283 (186, 427)	<0.001^a^
Hospitalization in FY2009, n (%)	223 (8.5)	127 (8.5)	60 (9.5)	0.68^b^
Annual inpatient expenditures in FY2009 (¥1,000), Median (Q1, Q3)^f^	415 (151, 737)	410 (158, 911)	580 (296, 1,162)	0.039^a^
Total medical expenditures ≥ ¥1 million in FY2009, n (%)	91 (3.4)	58 (3.9)	35 (5.6)	0.048^b^

BMI: Body mass index; LDL: Low-density lipoprotein; Q1: The first quartile; Q3: The third quartile

^a^Kruskal–Wallis rank sum test

^b^Pearson’s Chi-squared test

^c^Poor glycemic control: HbA1c ≥ 7.0% or fasting blood glucose ≥ 140 mg/dL

^d^Hypertension: Systolic blood pressure ≥ 140 mmHg or diastolic blood pressure ≥ 90 mmHg or taking antihypertensive medication

^e^Hyper-LDL cholesterolemia: LDL cholesterol ≥ 120 mg/dL or those taking cholesterol-lowering medications

^f^Median (Q1, Q3) for annual inpatient expenditures was calculated among participants who were hospitalized in FY2009.

### Association between BMI and annual total medical expenditures

The associations between BMI categories and medical expenditures among men and women are presented in Table 2. In Model 1, which adjusted for age, the overweight and obesity groups had significantly higher annual total medical expenditures than the normal/underweight group in both sexes. This trend remained after adjustment for age, smoking status, glycemic control, hypertension, hyper-LDL cholesterolemia, and mean annual medical expenditures from FY2007 to FY2008. Specifically, men in the obesity group had significantly higher annual total medical expenditures than those in the normal/underweight group (exp(β) = 1.117, 95% CI: 1.023–1.221). Similarly, women in the obesity group had significantly higher annual total medical expenditures than those in the normal/underweight group (exp(β) = 1.157, 95% CI: 1.036–1.295).

**Table 2:** Association between BMI categories and annual total medical expenditures.

	Model 1		Model 2	
Characteristics	exp(β) (95% CI)	p-value	exp(β) (95% CI)	p-value
Male				
Overweight (ref: normal/underweight)	1.126 (1.070 to 1.186)	<0.001	1.044 (0.991 to 1.099)	0.11
Obesity (ref: normal/underweight)	1.296 (1.190 to 1.414)	<0.001	1.117 (1.023 to 1.221)	0.013
Age	1.017 (1.014 to 1.020)	<0.001	1.011 (1.008 to 1.014)	<0.001
Poor glycemic control^a^			1.152 (1.098 to 1.208)	<0.001
Current smoker			1.072 (1.021 to 1.125)	0.005
Hypertension^b^			1.146 (1.090 to 1.204)	<0.001
Hyper-LDL cholesterolemia^c^			1.037 (0.987 to 1.089)	0.15
Mean annual medical expenditures FY2007–FY2008 (¥1,000)			1.002 (1.002 to 1.002)	<0.001
Female				
Overweight (ref: normal/underweight)	1.130 (1.044 to 1.223)	0.003	1.043 (0.964 to 1.130)	0.29
Obesity (ref: normal/underweight)	1.374 (1.233 to 1.536)	<0.001	1.157 (1.036 to 1.295)	0.010
Age	1.026 (1.021 to 1.031)	<0.001	1.018 (1.014 to 1.023)	<0.001
Poor glycemic control^a^			1.159 (1.081 to 1.244)	<0.001
Current smoker			1.175 (1.059 to 1.308)	0.003
Hypertension^b^			1.123 (1.043 to 1.210)	0.002
Hyper-LDL cholesterolemia^c^			1.012 (0.930 to 1.100)	0.78
Mean annual medical expenditures FY2007–FY2008 (¥1,000)			1.002 (1.002 to 1.002)	<0.001

BMI: Body mass index; CI: Confidence interval; LDL: Low-density lipoprotein

^a^Poor glycemic control: HbA1c ≥ 7.0% or fasting blood glucose ≥ 140 mg/dL

^b^Hypertension: Systolic blood pressure ≥ 140 mmHg or diastolic blood pressure ≥ 90 mmHg or taking antihypertensive medication

^c^Hyper-LDL cholesterolemia: LDL cholesterol ≥ 120 mg/dL or those taking cholesterol-lowering medications

### Association between BMI and annual outpatient expenditures

The associations between BMI categories and annual outpatient expenditures in men and women are presented in Table 3. In multivariable-adjusted models, both overweight and obesity were associated with significantly higher outpatient expenditures in both men and women compared with the normal/underweight group (men: overweight, exp(β) = 1.053, 95% CI: 1.028–1.078; obesity, exp(β) = 1.126, 95% CI: 1.082–1.172; women: overweight, exp(β) = 1.075, 95% CI: 1.035–1.118; obesity, exp(β) = 1.155, 95% CI: 1.094–1.220).

**Table 3:** Association between BMI categories and annual outpatient expenditures.

	Model 1		Model 2	
Characteristics	exp(β) (95% CI)	p-value	exp(β) (95% CI)	p-value
Male				
Overweight (ref: normal/underweight)	1.128 (1.099 to 1.158)	<0.001	1.053 (1.028 to 1.078)	<0.001
Obesity (ref: normal/underweight)	1.301 (1.244 to 1.360)	<0.001	1.126 (1.082 to 1.172)	<0.001
Age	1.012 (1.011 to 1.014)	<0.001	1.005 (1.004 to 1.007)	<0.001
Poor glycemic control^a^			1.099 (1.076 to 1.123)	<0.001
Current smoker			0.991 (0.970 to 1.013)	0.44
Hypertension^b^			1.089 (1.065 to 1.114)	<0.001
Hyper-LDL cholesterolemia^c^			1.009 (0.987 to 1.032)	0.43
Mean annual medical expenditures FY2007–FY2008 (¥1,000)			1.002 (1.002 to 1.002)	<0.001
Female				
Overweight (ref: normal/underweight)	1.171 (1.118 to 1.226)	<0.001	1.075 (1.035 to 1.118)	<0.001
Obesity (ref: normal/underweight)	1.351 (1.268 to 1.440)	<0.001	1.155 (1.094 to 1.220)	<0.001
Age	1.020 (1.017 to 1.023)	<0.001	1.010 (1.008 to 1.013)	<0.001
Poor glycemic control^a^			1.170 (1.131 to 1.211)	<0.001
Current smoker			0.996 (0.946 to 1.049)	0.88
Hypertension^b^			1.095 (1.056 to 1.136)	<0.001
Hyper-LDL cholesterolemia^c^			1.052 (1.010 to 1.096)	0.015
Mean annual medical expenditures FY2007–FY2008 (¥1,000)			1.002 (1.002 to 1.002)	<0.001

BMI: Body mass index; CI: Confidence interval; LDL: Low-density lipoprotein

^a^Poor glycemic control: HbA1c ≥ 7.0% or fasting blood glucose ≥ 140 mg/dL

^b^Hypertension: Systolic blood pressure ≥ 140 mmHg or diastolic blood pressure ≥ 90 mmHg or taking antihypertensive medication

^c^Hyper-LDL cholesterolemia: LDL cholesterol ≥ 120 mg/dL or those taking cholesterol-lowering medications

### Association between BMI and annual inpatient expenditures

Table 4 shows the association between BMI categories and annual inpatient expenditures. Among men, no significant association was found between BMI categories and annual inpatient expenditures in either Model 1 (age-adjusted only) or Model 2 (multivariable-adjusted). In contrast, the overweight and obesity groups had higher odds of hospitalization than the normal/underweight group. In Model 1, the obesity group had significantly higher odds of hospitalization (odds ratio for no hospitalization = 0.779, 95% CI: 0.650–0.934; *p* = 0.007). In Model 2, although the overweight and obesity groups also showed higher odds of hospitalization, these associations were no longer statistically significant. Among women, the associations between BMI categories and both annual inpatient expenditures and the incidence of hospitalization were weaker than those observed in men and were not statistically significant.

**Table 4:** Association between BMI categories and annual inpatient expenditures.

	Model 1		Model 2	
Characteristics	exp(β) (95% CI)	p-value	exp(β) (95% CI)	p-value
Male				
Gamma regression (Inpatient expenditures > 0)				
Overweight (ref: normal/underweight)	1.024 (0.911 to 1.151)	0.70	0.969 (0.861 to 1.091)	0.61
Obesity (ref: normal/underweight)	1.015 (0.836 to 1.233)	0.88	0.929 (0.760 to 1.135)	0.47
Age	1.008 (1.001 to 1.015)	0.036	1.007 (0.999 to 1.015)	0.074
Poor glycemic control^a^			1.132 (1.013 to 1.264)	0.028
Current smoker			1.167 (1.043 to 1.306)	0.007
Hypertension^b^			1.263 (1.125 to 1.418)	<0.001
Hyper-LDL cholesterolemia^c^			1.160 (1.036 to 1.299)	0.010
Mean annual medical expenditures FY2007–FY2008 (¥1,000)			1.000 (1.000 to 1.000)	0.006
Logistic regression (No hospitalization = 1)				
Overweight (ref: normal/underweight)	0.897 (0.803 to 1.001)	0.052	0.916 (0.818 to 1.026)	0.13
Obesity (ref: normal/underweight)	0.779 (0.650 to 0.934)	0.007	0.831 (0.689 to 1.002)	0.052
Age	0.974 (0.967 to 0.981)	<0.001	0.976 (0.969 to 0.983)	<0.001
Poor glycemic control^a^			0.848 (0.764 to 0.941)	0.002
Current smoker			0.853 (0.767 to 0.948)	0.003
Hypertension^b^			0.943 (0.845 to 1.052)	0.29
Hyper-LDL cholesterolemia^c^			1.010 (0.907 to 1.125)	0.85
Mean annual medical expenditures FY2007–FY2008 (¥1,000)			0.999 (0.999 to 0.999)	<0.001
Female				
Gamma regression (Inpatient expenditures > 0)				
Overweight (ref: normal/underweight)	0.931 (0.737 to 1.177)	0.55	0.898 (0.710 to 1.136)	0.37
Obesity (ref: normal/underweight)	1.117 (0.817 to 1.526)	0.49	1.097 (0.794 to 1.516)	0.57
Age	1.015 (1.001 to 1.029)	0.038	1.013 (0.998 to 1.028)	0.085
Poor glycemic control^a^			0.775 (0.627 to 0.958)	0.018
Current smoker			1.428 (1.069 to 1.908)	0.016
Hypertension^b^			1.142 (0.908 to 1.437)	0.26
Hyper-LDL cholesterolemia^c^			0.888 (0.689 to 1.144)	0.36
Mean annual medical expenditures FY2007–FY2008 (¥1,000)			1.000 (1.000 to 1.001)	0.15
Logistic regression (No hospitalization = 1)				
Overweight (ref: normal/underweight)	0.977 (0.777 to 1.228)	0.84	1.074 (0.849 to 1.358)	0.55
Obesity (ref: normal/underweight)	0.755 (0.557 to 1.023)	0.070	0.890 (0.648 to 1.221)	0.47
Age	0.956 (0.943 to 0.969)	<0.001	0.958 (0.944 to 0.972)	<0.001
Poor glycemic control^a^			0.702 (0.571 to 0.864)	<0.001
Current smoker			0.642 (0.480 to 0.859)	0.003
Hypertension^b^			0.831 (0.664 to 1.040)	0.11
Hyper-LDL cholesterolemia^c^			0.988 (0.766 to 1.273)	0.92
Mean annual medical expenditures FY2007–FY2008 (¥1,000)			0.999 (0.999 to 1.000)	<0.001

BMI: Body mass index; CI: Confidence interval; LDL: Low-density lipoprotein

^a^Poor glycemic control: HbA1c ≥ 7.0% or fasting blood glucose ≥ 140 mg/dL

^b^Hypertension: Systolic blood pressure ≥ 140 mmHg or diastolic blood pressure ≥ 90 mmHg or taking antihypertensive medication

^c^Hyper-LDL cholesterolemia: LDL cholesterol ≥ 120 mg/dL or those taking cholesterol-lowering medications

### Association between BMI categories and annual total medical expenditures at quantile points

Table 5 shows the results of the quantile regression analysis examining the association between annual total medical expenditures and BMI categories. The characteristics of participants by quantiles of medical expenditures in FY2009 are presented in S1 and S2 Tables.

**Table 5:** Quantile regression analysis of annual total medical expenditures attributable to overweight or obesity.

	10th percentile		25th percentile		50th percentile		75th percentile		90th percentile	
	β (95% CI)	p-value	β (95% CI)	p-value	β (95% CI)	p-value	β (95% CI)	p-value	β (95% CI)	p-value
Male										
Overweight (ref: normal/underweight)	4.7 (−3.1 to 12.5)	0.23	5.0 (1.3 to 8.7)	0.008	5.4 (2.6 to 8.3)	<0.001	8.4 (3.1 to 13.6)	0.002	13.3 (−11.2 to 37.9)	0.29
Obesity (ref: normal/underweight)	13.7 (2.4 to 25.0)	0.017	9.3 (4.4 to 14.1)	<0.001	12.2 (6.5 to 17.9)	<0.001	19.3 (12.6 to 26.0)	<0.001	28.1 (−30.7 to 87.0)	0.35
Age	−1.8 (−2.2 to −1.4)	<0.001	−0.4 (−0.7 to −0.2)	<0.001	0.2 (0.0 to 0.4)	0.019	1.2 (0.8 to 1.5)	<0.001	4.2 (2.6 to 5.8)	<0.001
Poor glycemic control^a^	6.5 (0.4 to 12.6)	0.037	7.6 (4.4 to 10.9)	<0.001	10.7 (8.1 to 13.3)	<0.001	16.6 (12.1 to 21.2)	<0.001	43.7 (18.9 to 68.6)	<0.001
Current smoker	−8.1 (−14.7 to −1.5)	0.016	−3.9 (−7.2 to −0.7)	0.017	−0.3 (−2.9 to 2.3)	0.82	2.7 (−1.9 to 7.3)	0.25	37.7 (11.6 to 63.8)	0.005
Hypertension^b^	0.8 (−5.8 to 7.3)	0.82	4.7 (1.3 to 8.1)	0.007	3.9 (1.2 to 6.6)	0.005	4.7 (−0.2 to 9.6)	0.059	6.1 (−18.4 to 30.6)	0.63
Hyper-LDL cholesterolemia^c^	−1.9 (−8.1 to 4.2)	0.54	1.0 (−2.4 to 4.5)	0.56	0.7 (−2.1 to 3.4)	0.64	−0.9 (−5.6 to 3.8)	0.72	−0.1 (−23.9 to 23.7)	>0.99
Mean annual medical expenditures FY2007–FY2008 (¥1,000)	0.4 (0.4 to 0.5)	<0.001	0.8 (0.8 to 0.8)	<0.001	0.9 (0.9 to 0.9)	<0.001	1.1 (1.0 to 1.1)	<0.001	1.4 (1.3 to 1.5)	<0.001
Change in annual medical expenditures from FY2007 to FY2008 (¥1,000)	0.2 (0.2 to 0.3)	<0.001	0.4 (0.4 to 0.4)	<0.001	0.5 (0.5 to 0.5)	<0.001	0.5 (0.5 to 0.5)	<0.001	0.6 (0.5 to 0.7)	<0.001
Female										
Overweight (ref: normal/underweight)	8.5 (−10.9 to 27.9)	0.39	1.7 (−3.9 to 7.3)	0.55	7.3 (1.1 to 13.4)	0.021	8.1 (−1.7 to 17.9)	0.10	−13.6 (−38.0 to 10.7)	0.27
Obesity (ref: normal/underweight)	11.6 (−8.4 to 31.6)	0.26	2.0 (−6.1 to 10.1)	0.63	5.2 (−3.7 to 14.1)	0.25	18.4 (1.8 to 34.9)	0.029	58.3 (−23.6 to 140.3)	0.16
Age	1.1 (0.1 to 2.1)	0.036	0.6 (0.2 to 0.9)	<0.001	0.7 (0.4 to 1.1)	<0.001	1.3 (0.7 to 1.9)	<0.001	4.4 (2.5 to 6.3)	<0.001
Poor glycemic control^a^	15.5 (−2.6 to 33.7)	0.093	15.7 (10.5 to 20.9)	<0.001	17.5 (12.2 to 22.8)	<0.001	24.1 (14.9 to 33.4)	<0.001	48.5 (16.4 to 80.6)	0.003
Current smoker	−13.2 (−30.9 to 4.5)	0.14	−1.6 (−9.6 to 6.4)	0.69	2.7 (−6.0 to 11.4)	0.55	3.6 (−8.5 to 15.7)	0.56	38.6 (−55.2 to 132.4)	0.42
Hypertension^b^	4.5 (−10.5 to 19.5)	0.55	8.3 (3.1 to 13.6)	0.002	1.4 (−3.9 to 6.8)	0.60	1.6 (−7.6 to 10.9)	0.73	19.1 (−15.3 to 53.4)	0.28
Hyper-LDL cholesterolemia^c^	9.0 (−5.0 to 23.0)	0.21	9.0 (2.1 to 15.9)	0.011	3.9 (−1.6 to 9.4)	0.16	−2.6 (−12.1 to 6.9)	0.59	−13.0 (−40.9 to 14.9)	0.36
Mean annual medical expenditures FY2007–FY2008 (¥1,000)	0.5 (0.4 to 0.6)	<0.001	0.8 (0.7 to 0.8)	<0.001	0.9 (0.9 to 0.9)	<0.001	1.0 (1.0 to 1.1)	<0.001	1.2 (1.1 to 1.4)	<0.001
Change in annual medical expenditures from FY2007 to FY2008 (¥1,000)	0.2 (0.2 to 0.3)	<0.001	0.4 (0.3 to 0.4)	<0.001	0.5 (0.4 to 0.5)	<0.001	0.5 (0.5 to 0.5)	<0.001	0.6 (0.5 to 0.8)	<0.001

CI: Confidence interval; LDL: Low-density lipoprotein

^a^Poor glycemic control: HbA1c ≥ 7.0% or fasting blood glucose ≥ 140 mg/dL

^b^Hypertension: Systolic blood pressure ≥ 140 mmHg or diastolic blood pressure ≥ 90 mmHg or taking antihypertensive medication

^c^Hyper-LDL cholesterolemia: LDL cholesterol ≥ 120 mg/dL or those taking cholesterol-lowering medications

Among men, annual total medical expenditures were higher in the overweight and obesity groups than in the normal/underweight group across all quantiles, with significant differences at the 25th, 50th, and 75th percentiles. Among women, annual total medical expenditures were higher in the overweight and obesity groups than in the normal/underweight group across most quantiles, except at the 90th percentile.

**Fig 1:**
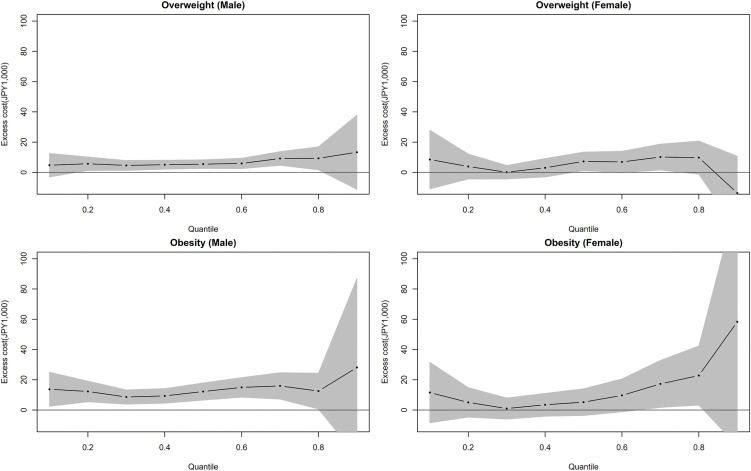
Distribution of adjusted excess medical expenditures in the overweight and obesity groups by percentile of annual total medical expenditures in FY2009. Top-left refers to the men who are overweight, top-right to women who are overweight, bottom-left to men with obesity, bottom-right to women with obesity. Dotted lines indicate the point estimates of adjusted excess medical expenditures, and gray shading indicates the 95% confidence intervals.

Among men, excess expenditures in the overweight and obesity groups were relatively similar across quantiles (Fig 1). Among women, excess expenditures associated with overweight showed little variation across most quantiles, whereas those associated with obesity tended to increase at higher quantiles, forming a J-shaped pattern (Fig 1).

### Association between BMI categories and the incidence of high medical expenditures

Table 6 presents the association between BMI categories and the incidence of high medical expenditures (annual total medical expenditures ≥ ¥1 million). Among men, in the age-adjusted model, the overweight and obesity groups had a higher risk of incurring high medical expenditures. However, in the multivariable-adjusted model, these associations were no longer significant. Among women, obesity was associated with a higher risk of high medical expenditures in both the age-adjusted model (OR = 1.970, 95% CI: 1.312–2.957; *p* = 0.001) and the multivariable-adjusted model (OR = 1.546, 95% CI: 1.007–2.375; *p* = 0.047).

**Table 6:** Association between BMI categories and annual total medical expenditures of ≥ ¥1 million.

	Model 1		Model 2	
	OR (95% CI)	p-value	OR (95% CI)	p-value
Male				
Overweight (ref: normal/underweight)	1.212 (1.032 to 1.424)	0.019	1.143 (0.967 to 1.350)	0.12
Obesity (ref: normal/underweight)	1.417 (1.086 to 1.849)	0.010	1.222 (0.929 to 1.608)	0.15
Age	1.040 (1.029 to 1.051)	<0.001	1.034 (1.023 to 1.046)	<0.001
Poor glycemic control^a^			1.295 (1.108 to 1.513)	0.001
Current smoker			1.344 (1.148 to 1.573)	<0.001
Hypertension^b^			1.325 (1.121 to 1.565)	<0.001
Hyper-LDL cholesterolemia^c^			1.024 (0.873 to 1.201)	0.77
Mean annual medical expenditures FY2007–FY2008 (¥1,000)			1.002 (1.001 to 1.002)	<0.001
Female				
Overweight (ref: normal/underweight)	1.153 (0.823 to 1.614)	0.41	0.992 (0.700 to 1.406)	0.96
Obesity (ref: normal/underweight)	1.970 (1.312 to 2.957)	0.001	1.546 (1.007 to 2.375)	0.047
Age	1.056 (1.035 to 1.078)	<0.001	1.047 (1.025 to 1.070)	<0.001
Poor glycemic control^a^			1.120 (0.828 to 1.516)	0.46
Current smoker			1.663 (1.091 to 2.534)	0.018
Hypertension^b^			1.395 (0.990 to 1.966)	0.057
Hyper-LDL cholesterolemia^c^			0.804 (0.563 to 1.147)	0.23
Mean annual medical expenditures FY2007–FY2008 (¥1,000)			1.002 (1.001 to 1.002)	<0.001

BMI: Body mass index; OR: Odds ratio; CI: Confidence interval; LDL: Low-density lipoprotein

a Poor glycemic control: HbA1c ≥ 7.0% or fasting blood glucose ≥ 140 mg/dL

b Hypertension: Systolic blood pressure ≥ 140 mmHg or diastolic blood pressure ≥ 90 mmHg or taking antihypertensive medication

^c^Hyper-LDL cholesterolemia: LDL cholesterol ≥ 120 mg/dL or those taking cholesterol-lowering medications

### Sensitivity analysis

The results of the sensitivity analysis, which included drinking and exercise as control variables in the multivariable-adjusted models, are presented in S3–S5 Tables. The gamma regression analysis showed higher annual total medical expenditures among men in the overweight and obesity groups than in the normal/underweight group, although the differences were not statistically significant. Among women, annual total medical expenditures were higher in the obesity group than in the normal/underweight group; however, this difference was not statistically significant (S3 Table).

Among men, annual outpatient expenditures were significantly higher in the overweight and obesity groups than in the normal/underweight group, consistent with the main analysis. Among women, the estimated effect size was slightly smaller than in the main analysis, and the significant association was no longer observed in the overweight group (S4 Table). No significant association between BMI categories and annual inpatient expenditures was observed among either men or women, consistent with the results observed in the main analysis (S5 Table).

The coefficient stability assessed using Oster’s method is shown in S6 Table. Among both men and women, in the models using annual total medical expenditures and total outpatient expenditures as outcomes, the δ values for the overweight and obesity groups were greater than 1, suggesting that the estimated coefficients were robust to unobserved confounding factors.

In the quantile regression analysis of the association between annual total medical expenditures and BMI categories, annual total medical expenditures were significantly higher in the overweight and obesity groups than in the normal/underweight group among men at the 25th, 50th, and 75th percentiles, consistent with the main analysis. Among women, a similar pattern was observed (S7 Table).

The results of the sensitivity analysis of the association between high medical expenditures and BMI categories are shown in S8 Table. In both sexes, the odds of incurring high medical expenditures in the obesity group were lower than those observed in the main analysis, and among women, the association was no longer significant.

## Discussion

In the present study, we found that, in both sexes, annual total medical expenditures were significantly higher in individuals with obesity, independent of hypertension, hyper-LDL cholesterolemia, and mean annual medical expenditures over the past two years. When analyses were stratified by outpatient and inpatient care, outpatient expenditures in the overweight and obesity groups were 5.3%–15.5% higher than those in the normal/underweight group in both men and women, and these differences were statistically significant. In contrast, no significant association was observed for inpatient expenditures.

Moreover, the quantile regression analysis showed that medical expenditures were generally higher in the overweight and obesity groups than in the normal/underweight group across quantiles. Among men, excess expenditures were relatively similar across quantiles, whereas among women, excess expenditures associated with overweight showed little variation across most quantiles, while those associated with obesity tended to increase at higher quantiles, forming a J-shaped pattern.

In addition, the incidence of high medical expenditures was higher in individuals with obesity, and this association was statistically significant among women. Collectively, these results suggest that medical expenditures were higher among both men and women with overweight or obesity, potentially reflecting suboptimally managed complications, such as diabetic nephropathy and cardiovascular disease.

The higher medical expenditures observed in this study among patients with diabetes and high BMI are consistent with those reported in previous studies in Japan and other countries [3,6–9,17,18]. A key strength of this study is its examination of the association between medical expenditures and BMI categories among individuals undergoing diabetes treatment, with a focus on distinguishing between overweight and obesity by sex. This approach is particularly important in Japan, where obesity is less prevalent than in Western countries [10], making it difficult to obtain sufficiently large sample sizes. To the best of our knowledge, such studies in Japan are limited. Previous studies in Japan have shown that individuals with a BMI ≥ 25 kg/m² have higher annual total medical expenditures than those with a BMI < 25 kg/m² [7]. In the present study, annual total medical expenditures were higher in the obesity group than in the overweight group. Further analyses separating outpatient and inpatient expenditures showed that outpatient expenditures were significantly higher in both the overweight and obesity groups, whereas no clear association with BMI category was observed for inpatient expenditures. These findings suggest that higher outpatient expenditures contribute substantially to the higher annual total medical expenditures observed among individuals with higher BMI.

With respect to annual medical expenditures of ≥ ¥1 million, a higher risk was observed in the obesity group. Although the association was not statistically significant among men, women with obesity showed a significantly elevated risk. However, since the available data did not include information on medical expenditures by disease or medication, the underlying reasons for the association between obesity and high medical expenditures among women could not be determined.

In addition, the association between BMI categories and annual total medical expenditures across percentiles showed that the increase in excess expenditures around the 90th percentile among men was less pronounced than that reported in a previous study [8]. At low-to-moderate expenditure levels, the overweight group had annual expenditures approximately ¥5,000 (approximately US$53 in 2009) higher than those of the normal/underweight group, while the obesity group had expenditures approximately ¥10,000 (approximately US$106 in 2009) higher. In contrast, among women in the obesity group, excess expenditures appeared to increase in the high-expenditure range around the 90th percentile, consistent with the previous study [8], although estimates were imprecise.

The J-shaped pattern observed among women with obesity should be interpreted with caution. The significant association at the 75th percentile and the large but non-significant estimate at the 90th percentile (β = 58.3, *p* = 0.16) may reflect a limited number of high-cost cases whose clinical characteristics cannot be determined from the available data. Several mechanisms could contribute to this pattern, including uncontrolled complications requiring expensive interventions, obesity-related comorbidities, and reverse causality through weight-gaining medications prescribed for more severe diabetes. However, the wide confidence interval at the 90th percentile indicates substantial uncertainty. These findings indicate notable differences in the observed trends between men and women. Overall, the results support current recommendations to consider obesity when selecting treatment options [19], even from the perspective of medical expenditures.

Weight control interventions for patients with diabetes include diet, exercise, behavioral therapy, pharmacotherapy, and surgery for severe obesity [20,21]. However, exercise therapy is not as widely implemented as dietary guidance. Several factors contribute to this underutilization, including the lack of additional reimbursement, a shortage of specialists, and limited time availability [22]. Another contributing factor is that working-age patients often work long hours, limiting their ability to make lifestyle modifications [23,24]. To address these barriers, improvements are needed in both the social environment surrounding patients with diabetes and the healthcare delivery system to better support weight control in patients with overweight or obesity.

Several limitations of this study should be considered when interpreting the findings. First, there is a risk of reverse causality, as insulin and insulin secretagogues can cause weight gain and obesity. Since data on specific medications were unavailable, the influence of these variables could not be directly controlled. This reverse causality may lead to an overestimation of the association between obesity and medical expenditures, particularly at higher quantiles where expenditures are greater, potentially contributing to the observed J-curve pattern. This differential bias may be especially relevant to the J-curve pattern observed among women with obesity, warranting caution in interpreting the magnitude of excess expenditures at the 75th and 90th percentiles. On the other hand, mean annual medical expenditures for FY2007–FY2008 and glycemic control status, which were included as covariates, may partially account for the influence of reverse causality. Furthermore, the sensitivity analysis using Oster’s method yielded δ substantially greater than 1, suggesting that even if variables such as medication types—which could directly account for reverse causality—were included, a reversal in expenditures between the obesity and normal/underweight groups would be unlikely.

Second, this study is a secondary analysis of data collected more than 10 years ago. Since 2009, when the data used in this study were collected, DPP-4 inhibitors, GLP-1 receptor agonists, SGLT2 inhibitors, and GIP/GLP-1 receptor agonists have become available [25]. The Japanese Diabetes Society’s treatment algorithm for type 2 diabetes now recommends weight-reducing agents, such as SGLT2 inhibitors, GLP-1 receptor agonists, and GIP/GLP-1 agonists, for patients with obesity [26]. Both the medical expenditures patterns for patients with diabetes and obesity and the susceptibility to medication-induced weight gain have likely changed since 2009. Although these newer agents are associated with weight loss, they are also more expensive. Therefore, analyses using more recent data are needed to determine whether their higher costs are offset by reductions in medical expenditures attributable to improved glycemic and weight control.

A further limitation is that well-known confounders, such as alcohol consumption, physical activity, and diet, were not included in the main analysis due to high rates of missing data. Sensitivity analyses incorporating these variables showed that while statistical significance changed owing to the reduced sample size, the direction of the associations remained largely unchanged. At a minimum, the finding that outpatient expenditures are higher among individuals with overweight and obesity appears robust. The coefficient stability analysis using Oster’s method further supports the robustness of the observed association between BMI categories and medical expenditures.

Finally, the study sample was limited to individuals who participated in the Specific Health Checkups. Participation rates were 20% for men and 27.3% for women among municipal insurers, and 33.4% for men and 20.2% for women among employee insurers [11]. Furthermore, hospitalized individuals and those residing in care facilities were excluded, meaning the sample represents a relatively healthy and health-conscious population. Therefore, the observed associations between BMI and medical expenditures may not be generalizable to hospitalized individuals, those in care facilities, or those with greater disease severity—groups who may incur annual medical expenditures of ≥ ¥1 million regardless of obesity level. Consequently, the association between obesity and high medical expenditures likely represents an underestimate of the true obesity–cost gradient in the broader population with diabetes.

## Conclusion

This study examined the association between BMI categories and medical expenditures by sex using data from Japan’s Specific Health Checkups and medical expenditure records from FY2007 to FY2009. The results showed that, in both men and women, the overweight (BMI ≥ 25 kg/m² and < 30 kg/m²) and obesity (BMI ≥ 30 kg/m²) groups had higher annual total medical expenditures than the normal/underweight group (BMI < 25 kg/m²), with the obesity group exhibiting significantly higher expenditures. When expenditures were examined separately by care type, outpatient expenditures (including medication costs) were significantly higher in both the overweight and obesity groups, whereas no significant differences were observed for inpatient expenditures.

Among men, excess expenditures associated with overweight and obesity were similar across quantiles of annual total medical expenditures. In contrast, among women, excess expenditures associated with obesity tended to be higher in the upper quantiles. In addition, the risk of incurring annual medical expenditures of ≥ ¥1 million was higher in women with obesity.

## Supporting information

S1 Table Participant characteristics by quantiles of annual medical expenditures in FY2009 (Male). (DOCX)

S2 Table Participant characteristics by quantiles of annual medical expenditures in FY2009 (Female). (DOCX)

S3 Table Sensitivity analysis of the association between BMI categories and annual total medical expenditures. (DOCX)

S4 Table Sensitivity analysis of the association between BMI categories and annual outpatient expenditures. (DOCX)

S5 Table Sensitivity analysis of the association between BMI categories and annual inpatient expenditures. (DOCX)

S6 Table Coefficient stability of gamma regression models. (DOCX)

S7 Table Sensitivity analysis for quantile regression analysis. (DOCX)

S8 Table Sensitivity analysis of the association between annual medical expenditures of ≥ ¥1 million and BMI categories. (DOCX)
