# Supplementary material for: Association between obesity and medical expenditures among Japanese adults treated for diabetes: A secondary analysis
Source: PLoS One. 2026 May 19;21(5):e0349416. doi: 10.1371/journal.pone.0349416 (PMC13186383; doi:10.1371/journal.pone.0349416)
Supplement: S1 Table — (DOCX) [file pone.0349416.s001.docx]

**S1 Table. Participant characteristics by quantiles of annual medical expenditures in FY2009 (Male)**

| **Characteristic** | **0-10 percentile**  N = 1,584 | **-25 percentile**  N = 2,375 | **-50 percentile**  N = 3,959 | **-75 percentile**  N = 3,958 | **-90 percentile**  N = 2,373 | **-100 percentile**  N = 1,584 | **p-value** |
| --- | --- | --- | --- | --- | --- | --- | --- |
| BMI, n (%) |  |  |  |  |  |  | <0.001^a^ |
| normal/underweight | 993 (63) | 1,476 (62) | 2,251 (57) | 2,068 (52) | 1,216 (51) | 804 (51) |  |
| overweight | 492 (31) | 748 (31) | 1,389 (35) | 1,472 (37) | 859 (36) | 614 (39) |  |
| obesity | 99 (6.3) | 151 (6.4) | 319 (8.1) | 418 (11) | 298 (13) | 166 (10) |  |
| Age, years, Median (Q1, Q3) | 59 (52 – 63) | 57 (51 – 61) | 58 (52 – 62) | 58 (53 – 62) | 58 (53 – 63) | 59 (54 – 65) | <0.001^b^ |
| Smoking, n (%) | 809 (51) | 1,167 (49) | 1,827 (46) | 1,739 (44) | 968 (41) | 673 (42) | <0.001 ^a^ |
| Poor glycemic control^c^, n (%) | 791 (50) | 1,203 (51) | 2,114 (53) | 2,191 (55) | 1,391 (59) | 929 (59) | <0.001 ^a^ |
| Hypertension^d^, n (%) | 787 (50) | 1,055 (44) | 2,124 (54) | 2,359 (60) | 1,523 (64) | 1,062 (67) | <0.001 ^a^ |
| Hyper-LDL cholesterolemia^e^, n (%) | 989 (62) | 1,472 (62) | 2,496 (63) | 2,547 (64) | 1,533 (65) | 984 (62) | 0.22 ^a^ |
| Mean annual medical expenditures FY2007–FY2008 (\1,000), Median (Q1, Q3) | 135 (55 – 253) | 131 (91 – 191) | 188 (142 – 236) | 273 (212 – 339) | 403 (302 – 504) | 382 (235 – 603) | <0.001 ^b^ |
| Change in annual medical expenditures from FY2007 to FY2008 (\1,000), Median (Q1, Q3) | 3 (-32 – 55) | 5 (-35 – 53) | 13 (-27 – 70) | 19 (-35 – 88) | 22 (-52 – 98) | 21 (-54 – 112) | <0.001 ^b^ |

BMI: Body mass index; Q1: The first quartile; Q3: The third quartile; LDL: Low-density lipoprotein

^a^ Pearson's Chi-squared test

^b^ Kruskal–Wallis rank sum test

^c^ Poor glycemic control: HbA1c ≥ 7.0% or fasting blood glucose ≥ 140 mg/dL

^d^ Hypertension: Systolic blood pressure ≥ 140 mmHg or diastolic blood pressure ≥ 90 mmHg or taking antihypertensive medication

^e^ Hyper-LDL cholesterolemia: LDL cholesterol ≥ 120 mg/dL or those taking cholesterol-lowering medications
