# Supplementary material for: Association between obesity and medical expenditures among Japanese adults treated for diabetes: A secondary analysis
Source: PLoS One. 2026 May 19;21(5):e0349416. doi: 10.1371/journal.pone.0349416 (PMC13186383; doi:10.1371/journal.pone.0349416)
Supplement: S2 Table — (DOCX) [file pone.0349416.s002.docx]

**S2 Table. Participant characteristics by quantiles of annual medical expenditures in FY2009 (Female)**

| **Characteristic** | **0-10 percentile** N = 476 | **-25 percentile**  N = 714 | **-50 percentile**  N = 1,190 | **-75 percentile**  N = 1,190 | **-90 percentile**  N = 714 | **-100 percentile**  N = 476 | **p-value** |
| --- | --- | --- | --- | --- | --- | --- | --- |
| BMI, n (%) |  |  |  |  |  |  | <0.001^a^ |
| normal/underweight | 324 (68) | 443 (62) | 676 (57) | 608 (51) | 360 (50) | 227 (48) |  |
| overweight | 112 (24) | 198 (28) | 370 (31) | 405 (34) | 243 (34) | 164 (34) |  |
| obesity | 40 (8.4) | 73 (10) | 144 (12) | 177 (15) | 111 (16) | 85 (18) |  |
| Age, years, Median (Q1, Q3) | 58 (50 – 61) | 58 (52 – 61) | 59 (55 – 64) | 60 (55 – 65) | 61 (55 – 67) | 62 (56 – 69) | <0.001^b^ |
| Smoking, n (%) | 71 (15) | 109 (15) | 131 (11) | 142 (12) | 74 (10) | 74 (16) | 0.004 ^a^ |
| Poor glycemic control^c^, n (%) | 164 (34) | 256 (36) | 557 (47) | 598 (50) | 400 (56) | 261 (55) | <0.001 ^a^ |
| Hypertension^d^, n (%) | 206 (43) | 314 (44) | 631 (53) | 753 (63) | 472 (66) | 335 (70) | <0.001 ^a^ |
| Hyper-LDL cholesterolemia^e^, n (%) | 326 (68) | 528 (74) | 951 (80) | 949 (80) | 563 (79) | 371 (78) | <0.001 ^a^ |
| Mean annual medical expenditures FY2007–FY2008 (\1,000), Median (Q1, Q3) | 115 (25 – 244) | 135 (85 – 190) | 202 (156 – 252) | 296 (233 – 365) | 424 (314 – 525) | 463 (285 – 702) | <0.001 ^b^ |
| Change in annual medical expenditures from FY2007 to FY2008 (\1,000), Median (Q1, Q3) | 1 (-25 – 34) | 3 (-34 – 48) | 7 (-38 – 60) | 14 (-41 – 83) | 21 (-50 – 107) | 20 (-57 – 137) | <0.001 ^b^ |

BMI: Body mass index; Q1: The first quartile; Q3: The third quartile; LDL: Low-density lipoprotein

^a^ Pearson's Chi-squared test

^b^ Kruskal–Wallis rank sum test

^c^ Poor glycemic control: HbA1c ≥ 7.0% or fasting blood glucose ≥ 140 mg/dL

^d^ Hypertension: Systolic blood pressure ≥ 140 mmHg or diastolic blood pressure ≥ 90 mmHg or taking antihypertensive medication

^e^ Hyper-LDL cholesterolemia: LDL cholesterol ≥ 120 mg/dL or those taking cholesterol-lowering medications
