# Supplementary material for: Association between obesity and medical expenditures among Japanese adults treated for diabetes: A secondary analysis
Source: PLoS One. 2026 May 19;21(5):e0349416. doi: 10.1371/journal.pone.0349416 (PMC13186383; doi:10.1371/journal.pone.0349416)
Supplement: S3 Table — (DOCX) [file pone.0349416.s003.docx]

**S3 Table. Sensitivity analysis of the association between BMI categories and annual total medical expenditures**

|  | **Characteristic** | **exp(β)** **(95% CI)** | **p-value** |
| --- | --- | --- | --- |
| Male |  |  |  |
|  | Overweight (ref: normal/underweight) | 1.042 (0.976 to 1.114) | 0.22 |
|  | Obesity (ref: normal/underweight) | 1.078 (0.962 to 1.211) | 0.20 |
|  | Age | 1.010 (1.006 to 1.015) | <0.001 |
|  | Poor glycemic control^a^ | 1.151 (1.082 to 1.224) | <0.001 |
|  | Current smoker | 1.067 (1.002 to 1.137) | 0.042 |
|  | Hypertension^b^ | 1.149 (1.078 to 1.226) | <0.001 |
|  | Hyper-LDL cholesterolemia^c^ | 1.068 (1.002 to 1.138) | 0.043 |
|  | Mean annual medical expenditures FY2007–FY2008 (\1,000) | 1.002 (1.001 to 1.002) | <0.001 |
|  | Physical activity^d^ | 0.980 (0.916 to 1.050) | 0.57 |
|  | Drinking status^e^ | 0.945 (0.889 to 1.006) | 0.075 |
| Female |  |  |  |
|  | Overweight (ref: normal/underweight) | 0.977 (0.891 to 1.070) | 0.61 |
|  | Obesity (ref: normal/underweight) | 1.055 (0.927 to 1.204) | 0.42 |
|  | Age | 1.014 (1.008 to 1.020) | <0.001 |
|  | Poor glycemic control^a^ | 1.028 (0.947 to 1.114) | 0.51 |
|  | Current smoker | 1.151 (1.009 to 1.319) | 0.035 |
|  | Hypertension^b^ | 1.113 (1.019 to 1.215) | 0.016 |
|  | Hyper-LDL cholesterolemia^c^ | 0.974 (0.881 to 1.075) | 0.61 |
|  | Mean annual medical expenditures FY2007–FY2008 (\1,000) | 1.002 (1.001 to 1.002) | <0.001 |
|  | Physical activity^d^ | 0.991 (0.907 to 1.083) | 0.84 |
|  | Drinking status^e^ | 0.942 (0.806 to 1.108) | 0.45 |

BMI: Body mass index; CI: Confidence interval; LDL: Low-density lipoprotein

^a^ Poor glycemic control: HbA1c ≥ 7.0% or fasting blood glucose ≥ 140 mg/dL

^b^ Hypertension: Systolic blood pressure ≥ 140 mmHg or diastolic blood pressure ≥ 90 mmHg or taking antihypertensive medication

^c^ Hyper-LDL cholesterolemia: LDL cholesterol ≥ 120 mg/dL or those taking cholesterol-lowering medications

^d^ Physical activity: Light sweaty exercise for at least 30 min at a time, at least 2 days a week for at least 1 year.

^e^ Drinking status: drinking alcohol occasionally or daily, and drinking more than one cup of sake per day
