# Supplementary material for: Association between obesity and medical expenditures among Japanese adults treated for diabetes: A secondary analysis
Source: PLoS One. 2026 May 19;21(5):e0349416. doi: 10.1371/journal.pone.0349416 (PMC13186383; doi:10.1371/journal.pone.0349416)
Supplement: S4 Table — (DOCX) [file pone.0349416.s004.docx]

**S4 Table. Sensitivity analysis of the association between BMI categories and annual outpatient expenditures**

|  | **Characteristic** | **exp(β)** **(95% CI)** | **p-value** |
| --- | --- | --- | --- |
| Male |  |  |  |
|  | Overweight (ref: normal/underweight) | 1.048 (1.017 to 1.081) | 0.002 |
|  | Obesity (ref: normal/underweight) | 1.122 (1.065 to 1.183) | <0.001 |
|  | Age | 1.004 (1.002 to 1.007) | <0.001 |
|  | Poor glycemic control^a^ | 1.080 (1.050 to 1.111) | <0.001 |
|  | Current smoker | 0.980 (0.952 to 1.009) | 0.17 |
|  | Hypertension^b^ | 1.081 (1.049 to 1.113) | <0.001 |
|  | Hyper-LDL cholesterolemia^c^ | 1.011 (0.982 to 1.041) | 0.47 |
|  | Mean annual medical expenditures FY2007–FY2008 (\1,000) | 1.002 (1.002 to 1.002) | <0.001 |
|  | Physical activity^d^ | 1.014 (0.983 to 1.046) | 0.39 |
|  | Drinking status^e^ | 0.980 (0.953 to 1.008) | 0.16 |
| Female |  |  |  |
|  | Overweight (ref: normal/underweight) | 1.000 (0.957 to 1.045) | 0.99 |
|  | Obesity (ref: normal/underweight) | 1.069 (1.004 to 1.139) | 0.037 |
|  | Age | 1.006 (1.003 to 1.009) | <0.001 |
|  | Poor glycemic control^a^ | 1.073 (1.032 to 1.116) | <0.001 |
|  | Current smoker | 1.006 (0.945 to 1.073) | 0.85 |
|  | Hypertension^b^ | 1.087 (1.042 to 1.134) | <0.001 |
|  | Hyper-LDL cholesterolemia^c^ | 1.001 (0.953 to 1.050) | 0.98 |
|  | Mean annual medical expenditures FY2007–FY2008 (\1,000) | 1.002 (1.002 to 1.002) | <0.001 |
|  | Physical activity^d^ | 1.003 (0.962 to 1.047) | 0.87 |
|  | Drinking status^e^ | 0.946 (0.878 to 1.021) | 0.15 |

BMI: Body mass index; CI: Confidence interval; LDL: Low-density lipoprotein

^a^ Poor glycemic control: HbA1c ≥ 7.0% or fasting blood glucose ≥ 140 mg/dL

^b^ Hypertension: Systolic blood pressure ≥ 140 mmHg or diastolic blood pressure ≥ 90 mmHg or taking antihypertensive medication

^c^ Hyper-LDL cholesterolemia: LDL cholesterol ≥ 120 mg/dL or those taking cholesterol-lowering medications

^d^ Physical activity: Light sweaty exercise for at least 30 min at a time, at least 2 days a week for at least 1 year.

^e^ Drinking status: drinking alcohol occasionally or daily, and drinking more than one cup of sake per day
