# Supplementary material for: Association between obesity and medical expenditures among Japanese adults treated for diabetes: A secondary analysis
Source: PLoS One. 2026 May 19;21(5):e0349416. doi: 10.1371/journal.pone.0349416 (PMC13186383; doi:10.1371/journal.pone.0349416)
Supplement: S5 Table — (DOCX) [file pone.0349416.s005.docx]

**S5 Table. Sensitivity analysis of the association between BMI categories and annual inpatient expenditures**

|  | **Characteristic** | **exp(β)** **(95% CI)** | **p-value** |
| --- | --- | --- | --- |
| Male |  |  |  |
| Gamma regression (expenditures>0) |  |  |  |
|  | Overweight (ref: normal/underweight) | 1.032 (0.896 to 1.188) | 0.66 |
|  | Obesity (ref: normal/underweight) | 0.873 (0.679 to 1.124) | 0.29 |
|  | Age | 1.005 (0.995 to 1.015) | 0.31 |
|  | Poor glycemic control^a^ | 1.128 (0.986 to 1.290) | 0.079 |
|  | Current smoker | 1.148 (0.998 to 1.320) | 0.053 |
|  | Hypertension^b^ | 1.319 (1.148 to 1.515) | <0.001 |
|  | Hyper-LDL cholesterolemia^c^ | 1.250 (1.087 to 1.437) | 0.002 |
|  | Mean annual medical expenditures FY2007–FY2008 (\1,000) | 1.000 (1.000 to 1.000) | 0.20 |
|  | Physical activity^d^ | 0.865 (0.747 to 1.001) | 0.051 |
|  | Drinking status^e^ | 0.971 (0.849 to 1.112) | 0.67 |
| Logistic regression (no hospitalization = 1) |  |  |  |
|  | Overweight (ref: normal/underweight) | 0.924 (0.801 to 1.065) | 0.27 |
|  | Obesity (ref: normal/underweight) | 0.913 (0.714 to 1.169) | 0.47 |
|  | Age | 0.976 (0.966 to 0.985) | <0.001 |
|  | Poor glycemic control^a^ | 0.827 (0.725 to 0.944) | 0.005 |
|  | Current smoker | 0.843 (0.736 to 0.966) | 0.014 |
|  | Hypertension^b^ | 0.991 (0.862 to 1.140) | 0.90 |
|  | Hyper-LDL cholesterolemia^c^ | 0.942 (0.821 to 1.082) | 0.40 |
|  | Mean annual medical expenditures FY2007–FY2008 (\1,000) | 0.999 (0.999 to 0.999) | <0.001 |
|  | Physical activity^d^ | 1.001 (0.865 to 1.159) | 0.98 |
|  | Drinking status^e^ | 1.117 (0.977 to 1.277) | 0.11 |
| Female |  |  |  |
| Gamma regression (expenditures>0) |  |  |  |
|  | Overweight (ref: normal/underweight) | 0.946 (0.713 to 1.256) | 0.70 |
|  | Obesity (ref: normal/underweight) | 1.165 (0.785 to 1.729) | 0.45 |
|  | Age | 1.016 (0.998 to 1.035) | 0.084 |
|  | Poor glycemic control^a^ | 0.732 (0.577 to 0.928) | 0.010 |
|  | Current smoker | 1.165 (0.756 to 1.797) | 0.49 |
|  | Hypertension^b^ | 0.944 (0.707 to 1.260) | 0.69 |
|  | Hyper-LDL cholesterolemia^c^ | 0.978 (0.733 to 1.305) | 0.88 |
|  | Mean annual medical expenditures FY2007–FY2008 (\1,000) | 1.000 (1.000 to 1.001) | 0.52 |
|  | Physical activity^d^ | 0.816 (0.630 to 1.058) | 0.12 |
|  | Drinking status^e^ | 1.028 (0.572 to 1.848) | 0.93 |
| Logistic regression (no hospitalization = 1) |  |  |  |
|  | Overweight (ref: normal/underweight) | 1.024 (0.774 to 1.356) | 0.87 |
|  | Obesity (ref: normal/underweight) | 1.005 (0.671 to 1.506) | 0.98 |
|  | Age | 0.955 (0.937 to 0.973) | <0.001 |
|  | Poor glycemic control^a^ | 0.809 (0.630 to 1.039) | 0.10 |
|  | Current smoker | 0.618 (0.421 to 0.908) | 0.014 |
|  | Hypertension^b^ | 0.736 (0.557 to 0.973) | 0.031 |
|  | Hyper-LDL cholesterolemia^c^ | 1.124 (0.830 to 1.523) | 0.45 |
|  | Mean annual medical expenditures FY2007–FY2008 (\1,000) | 0.999 (0.999 to 1.000) | <0.001 |
|  | Physical activity^d^ | 0.947 (0.726 to 1.235) | 0.69 |
|  | Drinking status^e^ | 1.245 (0.725 to 2.138) | 0.43 |

BMI: Body mass index; CI: Confidence interval; LDL: Low-density lipoprotein

^a^ Poor glycemic control: HbA1c ≥ 7.0% or fasting blood glucose ≥ 140 mg/dL

^b^ Hypertension: Systolic blood pressure ≥ 140 mmHg or diastolic blood pressure ≥ 90 mmHg or taking antihypertensive medication

^c^ Hyper-LDL cholesterolemia: LDL cholesterol ≥ 120 mg/dL or those taking cholesterol-lowering medications

^d^ Physical activity: Light sweaty exercise for at least 30 min at a time, at least 2 days a week for at least 1 year.

^e^ Drinking status: drinking alcohol occasionally or daily, and drinking more than one cup of sake per day
