# Supplementary material for: Association between obesity and medical expenditures among Japanese adults treated for diabetes: A secondary analysis
Source: PLoS One. 2026 May 19;21(5):e0349416. doi: 10.1371/journal.pone.0349416 (PMC13186383; doi:10.1371/journal.pone.0349416)
Supplement: S6 Table — (DOCX) [file pone.0349416.s006.docx]

**S6 Table. Coefficient stability of gamma regression models**

|  |  | Outcome variables | | |
| --- | --- | --- | --- | --- |
| Male |  | Annual total | Outpatient | Inpatient |
| Coefficient for overweight | delta* | 12.6 | 11.4 | -9.42 |
|  | Uncontrolled coefficient | 0.189 | 0.19 | 0.0538 |
|  | Controlled coefficient | 0.174 | 0.173 | 0.0764 |
|  | Uncontrolled R-square | 0.00137 | 0.00145 | 0.0000618 |
| Coefficient for obesity | delta* | 6.63 | 5.92 | -4.69 |
|  | Uncontrolled coefficient | 0.337 | 0.333 | 0.0763 |
|  | Controlled coefficient | 0.278 | 0.27 | 0.154 |
|  | Uncontrolled R-square | 0.00159 | 0.00163 | 0.0000453 |
| Condition | Controlled R-square | 0.0448 | 0.0442 | 0.0134 |
|  | Max R-square | 0.06 | 0.06 | 0.02 |
|  | beta hat | 0 | 0 | 0 |
| Female |  |  |  |  |
| Coefficient for overweight | delta* | 5.77 | 5.07 | -4.43 |
|  | Uncontrolled coefficient | 0.23 | 0.231 | -0.0149 |
|  | Controlled coefficient | 0.17 | 0.168 | -0.0541 |
|  | Uncontrolled R-square | 0.00205 | 0.00213 | 0.0000054 |
| Coefficient for obesity | delta* | 14.1 | 10.6 | 10.1 |
|  | Uncontrolled coefficient | 0.321 | 0.311 | 0.151 |
|  | Controlled coefficient | 0.314 | 0.293 | 0.138 |
|  | Uncontrolled R-square | 0.00212 | 0.00206 | 0.000294 |
| Condition | Controlled R-square | 0.0838 | 0.0818 | 0.0189 |
|  | Max R-square | 0.11 | 0.11 | 0.025 |
|  | beta hat | 0 | 0 | 0 |

Max R-square was set to 1.3 $\times$ Controlled R-square based on the recommendation in Oster(2019).
