# Supplementary material for: Association between obesity and medical expenditures among Japanese adults treated for diabetes: A secondary analysis
Source: PLoS One. 2026 May 19;21(5):e0349416. doi: 10.1371/journal.pone.0349416 (PMC13186383; doi:10.1371/journal.pone.0349416)
Supplement: S7 Table — (DOCX) [file pone.0349416.s007.docx]

**S7 Table. Sensitivity analysis for quantile regression analysis**

|  | | **10th percentile** | | **25th percentile** | | **50th percentile** | | **75th percentile** | | **90th percentile** | |
| --- | --- | --- | --- | --- | --- | --- | --- | --- | --- | --- | --- |
|  | **Characteristic** | **β (95% CI)** | **p-value** | **β (95% CI)** | **p-value** | **β (95% CI)** | **p-value** | **β (95% CI)** | **p-value** | **β (95% CI)** | **p-value** |
| Male |  |  |  |  |  |  |  |  |  |  |  |
|  | Overweight  (ref: normal/underweight) | 7.1 (-3.0 to 17.2) | 0.17 | 5.5 (1.0 to 10.1) | 0.017 | 7.3 (3.6 to 11.1) | <0.001 | 11.7 (3.9 to 19.6) | 0.003 | 26.4 (-8.2 to 61.0) | 0.13 |
|  | Obesity  (ref: normal/underweight) | 27.7 (5.3 to 50.0) | 0.015 | 11.2 (4.5 to 17.8) | 0.001 | 13.1 (6.5 to 19.7) | <0.001 | 17.7 (6.3 to 29.0) | 0.002 | 23.1 (-37.0 to 83.2) | 0.45 |
|  | Age | -1.8 (-2.5 to -1.1) | <0.001 | -0.4 (-0.7 to -0.1) | 0.004 | 0.2 (-0.1 to 0.4) | 0.23 | 1.1 (0.6 to 1.6) | <0.001 | 4.7 (2.5 to 7.0) | <0.001 |
|  | Poor glycemic control^a^ | 5.1 (-4.6 to 14.8) | 0.30 | 5.5 (1.3 to 9.6) | 0.010 | 10.1 (6.7 to 13.5) | <0.001 | 17.2 (10.2 to 24.2) | <0.001 | 51.9 (18.9 to 84.9) | 0.002 |
|  | Current smoker | -14.6 (-24.0 to -5.2) | 0.002 | -5.3 (-9.6 to -1.0) | 0.016 | -1.0 (-4.5 to 2.5) | 0.58 | 3.1 (-4.1 to 10.2) | 0.40 | 45.7 (8.6 to 82.7) | 0.016 |
|  | Hypertension^b^ | -1.8 (-11.7 to 8.1) | 0.72 | 2.8 (-1.5 to 7.2) | 0.20 | 3.5 (0.0 to 7.1) | 0.050 | 2.7 (-4.7 to 10.1) | 0.47 | -11.3 (-46.0 to 23.3) | 0.52 |
|  | Hyper-LDL cholesterolemia^c^ | -3.6 (-12.9 to 5.7) | 0.45 | 2.6 (-1.8 to 7.0) | 0.25 | 2.8 (-0.8 to 6.5) | 0.13 | 3.5 (-3.5 to 10.6) | 0.33 | 14.5 (-17.5 to 46.6) | 0.37 |
|  | Mean annual medical expenditures FY2007–FY2008 (\1,000) | 0.5 (0.4 to 0.5) | <0.001 | 0.8 (0.8 to 0.8) | <0.001 | 0.9 (0.9 to 1.0) | <0.001 | 1.1 (1.0 to 1.1) | <0.001 | 1.4 (1.3 to 1.6) | <0.001 |
|  | Change in annual medical expenditures from FY2007 to FY2008 (\1,000) | 0.2 (0.2 to 0.3) | <0.001 | 0.4 (0.4 to 0.4) | <0.001 | 0.5 (0.5 to 0.5) | <0.001 | 0.5 (0.5 to 0.5) | <0.001 | 0.6 (0.4 to 0.8) | <0.001 |
|  | Physical activity^d^ | 10.0 (-0.6 to 20.7) | 0.065 | 2.4 (-2.3 to 7.2) | 0.31 | 0.1 (-4.0 to 4.2) | 0.97 | 0.3 (-8.4 to 9.0) | 0.95 | -18.7 (-51.4 to 14.1) | 0.26 |
|  | Drinking status^e^ | 0.6 (-9.5 to 10.6) | 0.91 | 1.2 (-3.0 to 5.4) | 0.57 | -0.5 (-3.9 to 3.0) | 0.78 | -1.8 (-8.9 to 5.3) | 0.62 | -22.1 (-55.0 to 10.9) | 0.19 |
| Female |  |  |  |  |  |  |  |  |  |  |  |
|  | Overweight  (ref: normal/underweight) | -3.1 (-27.8 to 21.5) | 0.80 | -1.9 (-9.7 to 5.9) | 0.63 | 6.5 (-1.2 to 14.1) | 0.10 | 0.0 (-11.0 to 11.0) | >0.99 | -4.0 (-42.8 to 34.8) | 0.84 |
|  | Obesity  (ref: normal/underweight) | -0.1 (-26.9 to 26.7) | >0.99 | 4.6 (-11.6 to 20.9) | 0.57 | 10.8 (-0.4 to 22.1) | 0.059 | 20.0 (5.5 to 34.4) | 0.007 | 41.1 (-14.0 to 96.2) | 0.14 |
|  | Age | 2.2 (0.6 to 3.9) | 0.008 | 0.9 (0.3 to 1.5) | 0.004 | 1.1 (0.6 to 1.5) | <0.001 | 1.8 (1.2 to 2.4) | <0.001 | 6.4 (4.0 to 8.9) | <0.001 |
|  | Poor glycemic control^a^ | 11.9 (-15.0 to 38.8) | 0.39 | 14.5 (6.9 to 22.1) | <0.001 | 18.2 (11.8 to 24.7) | <0.001 | 25.6 (15.9 to 35.3) | <0.001 | 43.4 (7.6 to 79.1) | 0.017 |
|  | Current smoker | 9.1 (-24.0 to 42.2) | 0.59 | 0.5 (-13.7 to 14.6) | 0.95 | 9.6 (0.1 to 19.0) | 0.047 | 4.3 (-13.0 to 21.7) | 0.62 | 39.5 (-43.2 to 122.2) | 0.35 |
|  | Hypertension^b^ | 19.5 (-2.3 to 41.3) | 0.079 | 8.2 (0.5 to 15.9) | 0.036 | 0.8 (-5.8 to 7.5) | 0.80 | 3.8 (-6.8 to 14.4) | 0.48 | 25.1 (-16.0 to 66.2) | 0.23 |
|  | Hyper-LDL cholesterolemia^c^ | 18.8 (-2.3 to 40.0) | 0.081 | 7.2 (-2.7 to 17.1) | 0.16 | 2.4 (-5.2 to 10.1) | 0.53 | -9.6 (-19.9 to 0.7) | 0.068 | -4.3 (-44.9 to 36.3) | 0.84 |
|  | Mean annual medical expenditures FY2007–FY2008 (\1,000) | 0.5 (0.4 to 0.6) | <0.001 | 0.8 (0.7 to 0.8) | <0.001 | 0.9 (0.9 to 1.0) | <0.001 | 1.0 (1.0 to 1.1) | <0.001 | 1.3 (1.1 to 1.4) | <0.001 |
|  | Change in annual medical expenditures from FY2007 to FY2008 (\1,000) | 0.3 (0.2 to 0.3) | <0.001 | 0.4 (0.3 to 0.4) | <0.001 | 0.5 (0.4 to 0.5) | <0.001 | 0.5 (0.5 to 0.5) | <0.001 | 0.6 (0.5 to 0.8) | <0.001 |
|  | Physical activity^d^ | 26.8 (5.9 to 47.8) | 0.012 | 6.4 (-1.3 to 14.1) | 0.10 | 5.8 (-0.7 to 12.3) | 0.078 | 1.3 (-9.2 to 11.9) | 0.81 | 0.1 (-50.0 to 50.2) | >0.99 |
|  | Drinking status^e^ | -12.7 (-45.0 to 19.7) | 0.44 | -2.2 (-17.9 to 13.4) | 0.78 | -3.2 (-12.4 to 6.0) | 0.50 | -9.6 (-29.0 to 9.9) | 0.33 | 3.0 (-57.7 to 63.8) | 0.92 |

CI: Confidence interval; LDL: Low-density lipoprotein

^a^ Poor glycemic control: HbA1c ≥ 7.0% or fasting blood glucose ≥ 140 mg/dL

^b^ Hypertension: Systolic blood pressure ≥ 140 mmHg or diastolic blood pressure ≥ 90 mmHg or taking antihypertensive medication

^c^ Hyper-LDL cholesterolemia: LDL cholesterol ≥ 120 mg/dL or those taking cholesterol-lowering medications

^d^ Physical activity: Light sweaty exercise for at least 30 min at a time, at least 2 days a week for at least 1 year.

^e^ Drinking status: drinking alcohol occasionally or daily, and drinking more than one cup of sake per day
