# Supplementary material for: Association between obesity and medical expenditures among Japanese adults treated for diabetes: A secondary analysis
Source: PLoS One. 2026 May 19;21(5):e0349416. doi: 10.1371/journal.pone.0349416 (PMC13186383; doi:10.1371/journal.pone.0349416)
Supplement: S8 Table — (DOCX) [file pone.0349416.s008.docx]

**S8 Table. Sensitivity analysis of the association between annual medical expenditures of** ≥**\1 million and BMI categories**

|  | **Characteristic** | **OR** **(95% CI)** | **p-value** |
| --- | --- | --- | --- |
| Male |  |  |  |
|  | Overweight  (ref: normal/underweight) | 1.187 (0.970 to 1.451) | 0.10 |
|  | Obesity  (ref: normal/underweight) | 1.038 (0.725 to 1.486) | 0.84 |
|  | Age | 1.034 (1.020 to 1.049) | <0.001 |
|  | Poor glycemic control^a^ | 1.292 (1.067 to 1.563) | 0.009 |
|  | Current smoker | 1.462 (1.203 to 1.777) | <0.001 |
|  | Hypertension^b^ | 1.318 (1.072 to 1.620) | 0.009 |
|  | Hyper-LDL cholesterolemia^c^ | 1.249 (1.020 to 1.529) | 0.031 |
|  | Mean annual medical expenditures FY2007–FY2008 (\1,000) | 1.001 (1.001 to 1.002) | <0.001 |
|  | Physical activity^d^ | 0.905 (0.732 to 1.120) | 0.36 |
|  | Drinking status^e^ | 0.870 (0.718 to 1.055) | 0.16 |
| Female |  |  |  |
|  | Overweight  (ref: normal/underweight) | 1.059 (0.706 to 1.590) | 0.78 |
|  | Obesity  (ref: normal/underweight) | 1.282 (0.739 to 2.223) | 0.38 |
|  | Age | 1.058 (1.029 to 1.087) | <0.001 |
|  | Poor glycemic control^a^ | 0.928 (0.646 to 1.332) | 0.68 |
|  | Current smoker | 1.516 (0.852 to 2.696) | 0.16 |
|  | Hypertension^b^ | 1.359 (0.900 to 2.054) | 0.14 |
|  | Hyper-LDL cholesterolemia^c^ | 0.809 (0.527 to 1.243) | 0.33 |
|  | Mean annual medical expenditures FY2007–FY2008 (\1,000) | 1.002 (1.001 to 1.002) | <0.001 |
|  | Physical activity^d^ | 0.939 (0.637 to 1.383) | 0.75 |
|  | Drinking status^e^ | 0.590 (0.234 to 1.490) | 0.26 |

OR: Odds ratio; CI: Confidence interval; LDL: Low-density lipoprotein

^a^ Poor glycemic control: HbA1c ≥ 7.0% or fasting blood glucose ≥ 140 mg/dL

^b^ Hypertension: Systolic blood pressure ≥ 140 mmHg or diastolic blood pressure ≥ 90 mmHg or taking antihypertensive medication

^c^ Hyper-LDL cholesterolemia: LDL cholesterol ≥ 120 mg/dL or those taking cholesterol-lowering medications

^d^ Physical activity: Light sweaty exercise for at least 30 min at a time, at least 2 days a week for at least 1 year.

^e^ Drinking status: drinking alcohol occasionally or daily, and drinking more than one cup of sake per day
